# Fourier Transform Infrared Spectroscopy as a Cancer Screening and Diagnostic Tool: A Review and Prospects

**DOI:** 10.3390/cancers12010115

**Published:** 2020-01-01

**Authors:** Kar-Yan Su, Wai-Leng Lee

**Affiliations:** School of Science, Monash University Malaysia, Subang Jaya 47500, Malaysia

**Keywords:** cancer, Fourier transform infrared spectroscopy, non-invasive diagnosis, screening, surgical management, treatment monitoring, clinical translation, extracellular vesicles

## Abstract

Infrared spectroscopy has long been used to characterize chemical compounds, but the applicability of this technique to the analysis of biological materials containing highly complex chemical components is arguable. However, recent advances in the development of infrared spectroscopy have significantly enhanced the capacity of this technique in analyzing various types of biological specimens. Consequently, there is an increased number of studies investigating the application of infrared spectroscopy in screening and diagnosis of various diseases. The lack of highly sensitive and specific methods for early detection of cancer has warranted the search for novel approaches. Being more simple, rapid, accurate, inexpensive, non-destructive and suitable for automation compared to existing screening, diagnosis, management and monitoring methods, Fourier transform infrared spectroscopy can potentially improve clinical decision-making and patient outcomes by detecting biochemical changes in cancer patients at the molecular level. Besides the commonly analyzed blood and tissue samples, extracellular vesicle-based method has been gaining popularity as a non-invasive approach. Therefore, infrared spectroscopic analysis of extracellular vesicles could be a useful technique in the future for biomedical applications. In this review, we discuss the potential clinical applications of Fourier transform infrared spectroscopic analysis using various types of biological materials for cancer. Additionally, the rationale and advantages of using extracellular vesicles in the spectroscopic analysis for cancer diagnostics are discussed. Furthermore, we highlight the challenges and future directions of clinical translation of the technique for cancer.

## 1. Introduction

Cancer is a major cause of death worldwide, accounting for a staggering estimated 9.6 million deaths in 2018 [1]. Globally, approximately one in three individuals will be diagnosed with cancer in his or her lifetime and one in six deaths is caused by cancer [1]. The poor survival rates reflect the fact that most patients are diagnosed at a stage which is not responsive to current treatments. Early cancer detection is crucial as the condition of a patient may be irreversible once the clinical symptoms appear. In fact, early detection of the disease and identification of at-risk individuals may delay or prevent further progression with suitable treatments and would greatly increase survival rate of patients. However, current screening and diagnostic methods including imaging techniques, normally detect cancer in late stage when tumor mass is visible and existing screening tests lack the necessary sensitivity and specificity at early stage of the disease [2].

To date, the gold standard for most cancer diagnosis is still the microscopic evaluation of stained tissue samples by pathologists, which is performed when cancerous or pre-cancerous lesions are observable and already contain significant genetic changes. Moreover, the use of the histopathological diagnosis is invasive, time consuming and has limited sensitivity as it depends heavily on the subjective judgement of pathologists which leads to intra- and inter-observer variations. Therefore, misdiagnosis with high false negative and false positive rates is common in tissue assessment [3]. In fact, approximately 10% of pathologic evaluation could not result in a firm diagnosis because either certain tumors are histologically similar or the tissue of origin could not be identified from the poorly differentiated cells [4]. The method also involves the complex process of histochemical staining techniques for the tissue samples, whereby the most commonly used hematoxylin and eosin (H&E) dyes are non-specific for cancer [5].

Biomarkers, which are defined as disease-related molecular changes in body fluids and tissues [6], are essential in facilitating screening and diagnosis to allow clinical interventions to begin as soon as possible. Conventional clinical analysis of blood samples for cancer diagnosis examines individual parameters which include tumor markers such as carcinoembryonic antigen (CEA), cancer antigen 15-3 (CA 15-3), prostate-specific antigen (PSA) and tissue polypeptide antigen (TPA). Nevertheless, these markers have low sensitivity and/or specificity [7,8,9]. Thus, the lack of highly specific and sensitive biomarkers for cancer as well as the limited number of non-invasive and cost-effective tests demand the discovery of novel biomarkers and diagnostic methods. Thus, combinations of biomarkers have been investigated to improve the current situation [10,11] and the use of multi-molecular biochemical analysis techniques such as Fourier transform infrared (FTIR) spectroscopy could support this purpose. As biochemical changes are preceded or accompanied with morphological alteration and symptomatic appearance correlated with disease progression or therapeutic treatment, the use of the vibrational spectroscopic technique can reveal these differences at molecular level and could serve as a screening and diagnostic tool [12]. In addition, instead of evaluating morphological differences as in current histopathology methods, the application of FTIR spectroscopy which analyses tissue samples at the molecular level before morphological changes arise without the need for staining has been investigated. This permits objective assessment of the samples which allows early detection and increases accuracy as well as minimizes discrepancies in the interpretation of pathologists.

Traditionally, FTIR spectroscopy has been used by chemists for the characterization of molecular structures. Nonetheless, the potential of the technique to analyze biological specimens as a cancer diagnostic tool has been recognized for decades [13]. The relatively simple and reproducible technique is reagent-free, non-destructive to samples and only require nanograms to micrograms of them with minimal preparation. The sensitivity of FTIR spectroscopy to chemical changes during the transition from normal to a pathological state or during treatment can lead to the identification of novel biomarkers associated with a disease [14]. Hence, FTIR spectroscopy is a robust tool with great potential for clinical application which extends beyond screening, diagnosis and prognosis of cancer to continuous monitoring of treatment response and disease progression or regression in personalized medicine.

Various types of biological materials including blood [15,16,17,18,19], tissues [4,20,21,22,23,24], extracellular vesicles (EVs) [25,26,27], urine [28], bladder wash [29], bile [30] and sputum [31,32] specimens have been studied using FTIR spectroscopy to develop better alternatives for cancer diagnosis and management. Blood and tissue samples are widely used in current clinical diagnostics for various diseases compared to other types of specimen. Nevertheless, the immense biological variability found in these complex biological specimens may mask specific spectral changes and hinder the identification of biomarkers. EVs have lately gained research interests due to their association with cancer. These vesicles have a diameter which ranges from 30–1000 nm and contain various biomolecules such as proteins, lipids and nucleic acids [33]. They are secreted by all cell types in the body and play a major role in intercellular communication. Intriguingly, EVs may reflect the condition of their originating cells and provide information of disease progression [34]. Therefore, EVs can be isolated from biofluids which are obtained non-invasively, such as urine, for FTIR spectroscopic analysis. This could remove the abundant uninformative and uncorrelated data to effectively identify biomarkers.

In this review, we discuss on the clinical applications of FTIR spectroscopy for cancer using biological materials ranging from the most commonly used blood and tissue specimens to the infrequently used urine and sputum samples. Additionally, the rationale and advantages of using EVs for FTIR spectroscopic analysis in cancer screening and diagnosis are discussed. Furthermore, we highlight the challenges and future directions in clinical translation of the technique for cancer.

## 2. Wavenumber Range and Computational Models in Fourier Transform Infrared Spectroscopic Analysis of Biological Specimens

FTIR spectroscopy detects biochemical compositions including nucleic acids, proteins, lipids and carbohydrates within biological samples by precisely identifying molecular conformations, bonding types, functional groups and intermolecular interactions of which the specimen is composed. As each molecule has a unique spectrum depending on the wavelength and quantity of infrared radiation being absorbed, IR spectroscopy produces a signature spectral fingerprint of absorbance peaks for multiplex parameters of genome, lipidome, proteome and metabolome in the examined sample. Essentially, the biochemical fingerprint changes are unique to the molecular alterations in specific diseases, providing valuable diagnostic information for each patient’s health status. As biological materials absorb energy in the mid-IR region (4000–400 cm^−1^ of the electromagnetic spectrum, the spectral regions typically measured for examining these specimens are the fingerprint region (1450–600 cm^−1^) as well as the amide I and II region (1700–1500 cm^−1^). Higher-wavenumber region (3500–2550 cm^−1^) is correlated to stretching vibrations including C–H, O–H, N–H and S–H, while lower-wavenumber regions are usually associated with bending and carbon skeletal fingerprint vibrations [35]. Table 1 shows the assignment of typical absorption bands identified in biological IR spectra [36,37,38].

By quantitatively measuring the vibrational modes [39] and analyze spectroscopic data to identify disease patterns with artificial intelligent systems, FTIR spectroscopy allows the advancement of next-generation clinical systems that could revolutionize disease diagnostics. Due to the molecular complexity of biological specimens, common techniques such as chemometrics which combine statistical and mathematical procedures are utilized to generate chemo-physical evidence from spectral data [40]. One of the chemometric techniques is principal component analysis (PCA), which is the most basic feature extraction unsupervised method based upon the analysis of feature variance within the full spectrum [36]. This technique has been coupled with FTIR spectroscopy in numerous studies for various applications in cancer, including cancer identification [41] and monitoring of chemotherapy efficacy [42]. Meanwhile, clustering unsupervised techniques including discriminant analysis (DA), hierarchical cluster analysis (HCA), support vector machines (SVM), artificial neural networks (ANN) and k-nearest neighbours (KNN) are applied to identify biological subtypes within a specimen. On the other hand, partial least squares (PLS) is the most extensively used supervised multivariate data analysis method which quantifies and estimates components in a sample [36]. Additionally, physics-based computational models have also been applied in FTIR spectroscopic analysis for cancer classification [43] and treatment monitoring to determine therapeutic efficacy [44,45].

## 3. Sensitivity, Specificity and Accuracy in Cancer Detection

The power of a test to differentiate patients from healthy individuals defines its accuracy and diagnostic value [46]. The characteristics which reflects the abilities of a test include accuracy, sensitivity and specificity. The accuracy of a test is defined as the ability to correctly distinguish the patient and healthy cases. The ideal diagnostic test would identify the cases with 100% accuracy. However, the accuracy of a test varies in different situations and for different diseases. Meanwhile, the sensitivity and specificity of a test are its ability to correctly determine the patient and healthy cases, respectively [47]. Even though these measures are usually regarded as fixed properties of a diagnostic test, they are subjected to multiple variations such as the severity of the disease under investigation and the population case mix [48]. In cancer diagnostic tests, current biomarkers used to detect the disease often have low sensitivity and/or specificity. For instance, the prostate-specific antigen (PSA) test has been used to detect prostate cancer. Although the test has a high specificity of approximately 87–95%, it has a much lower sensitivity which ranges from 33–59% [7].

FTIR spectroscopy has been shown to be a prospective novel diagnostic method for many different types of cancer by being able to distinguish cancer samples from normal ones at high sensitivity, specificity and accuracy. Sheng et al. have demonstrated the use of this technique in the diagnosis of leukemia [17] and gastric cancer [18] by analyzing serum samples. RNA/DNA and peak height ratios demonstrated high sensitivity and specificity of approximately 80–100% for the diagnosis of both cancers. Likewise, up to 98% of sensitivity and 100% of specificity have been reported by Backhaus et al. [15] in the analysis of serum samples for breast cancer diagnosis using FTIR spectroscopy as well as cluster analysis (CA) and artificial neural networks (ANN). Remarkably, these findings showed significant improvement over conventional clinical analysis of the tumor markers cancer antigen 15-3 (CA 15-3), carcinoembryonic antigen (CEA) and tissue polypeptide antigen (TPA) for breast cancer monitoring, which have only at most 60–70% of sensitivity and specificity [8,9,49]. Notably, the study also revealed the ability to distinguish breast cancer from other diseases such as Alzheimer’s disease, hepatitis C, coronary heart diseases as well as other types of cancer. On the other hand, Khanmohammadi et al. [4] applied attenuated total reflection (ATR)-FTIR microspectroscopy and chemometric techniques, such as CA, analysis of variance (ANOVA) and linear discriminant analysis (LDA), to diagnose colon cancer. Reproducible and clear differences between the spectra of cancer and normal colon tissues result in classification with high sensitivity, specificity and accuracy of 100%, 93.1% and 95.8%, respectively.

Furthermore, FTIR spectroscopy has been utilized for cancer diagnosis by analyzing other types of biological materials besides the commonly examined blood and tissue samples, such as urine [28], bladder wash [29], bile [30] and sputum [31,32] samples. Paraskevaidi et al. [28] have performed ATR-FTIR spectroscopic analysis of urine samples using classification models, including partial least squares discriminant analysis (PLS-DA), principal component analysis with support vector machines (PCA-SVM) and genetic algorithm with linear discriminant analysis (GA-LDA), for the non-invasive diagnostic test of ovarian and endometrial cancers. Urine samples from patients with ovarian or endometrial cancer and healthy controls were evaluated and achieved up to 100% of sensitivity, specificity as well as accuracy with the identified biomarkers for both types of cancers. Evidently, these results demonstrated the potential of the technique for improved diagnosis when compared to the most commonly used serum biomarker for ovarian cancer, cancer antigen 125 (CA-125), which has been found to be unacceptable for early-stage detection due to its low sensitivity of 50–60% and it is only elevated in approximately 60% of patients [50,51]. Apart from that, Lewis et al. [31] have applied FTIR spectroscopy combined with hierarchical cluster analysis (HCA) and principal component analysis (PCA) to examine sputum samples for the diagnosis of lung cancer. Likewise, Lewis et al. [31] obtained prominent significant wavenumbers which separate spectra between cancer and normal sputum samples. Interestingly, the spectral analysis showed that the wavenumbers were also able to differentiate lung cancer patients who had been previously diagnosed with breast cancer. The findings suggest that the techniques applied to sputum may have high sensitivity and specificity of greater than 80% for diagnosis using the small panel of significant wavenumbers, which compares more than favorably with current techniques of lung cancer detection. This enables the development of a non-invasive, cost-effective and high-throughput screening method for lung cancer.

Taken together, despite the sensitivity, specificity and accuracy of FTIR spectroscopy as a cancer diagnostic tool in these studies may be affected by factors such as the severity of the disease (e.g., stage and grade of cancer) in patient cases just as other diagnostic tests, the technique shows promising results for many different types of cancer when compared to existing diagnostic tests. Moreover, studies comparing different classification, stages and grades of cancer through FTIR analysis have been performed and are discussed in the following section. Although some studies may be preliminary and the sample size may be limited, these investigations demonstrated the strong potential of FTIR spectroscopy as a highly sensitive, specific and accurate cancer diagnostic tool that is valuable for further investigation and development.

## 4. Classification, Staging and Grading for Cancer Management

Effective cancer management requires accurate staging and grading of the disease to establish suitable treatments, predict clinical behavior of malignancies and facilitate interchange of precise information between clinicians. Cancer staging denotes the anatomic extent of the disease spread. The internationally accepted staging criteria for cancer, the tumor-node-metastasis (TNM) system, comprises: (1) tumor size and local growth (T); (2) extent of lymph node metastases (N); and (3) occurrence of distant metastases (M) [52]. The TNM system is used to classify cancer into stages from I to IV. Both clinical stage and pathologic stage can be assigned to the disease. Clinical stage is determined before initiation of treatment and depends on physical examinations, imaging studies and laboratory findings. Meanwhile, pathologic stage is established following histological examination of tissue and surgical exploration of disease spread [53]. Nonetheless, each cancer type has unique anatomical spread patterns and may require distinct TNM classification system. On the other hand, cancer grade is a subjective scoring by pathologists according to tumor histology and cytomorphology of tumor lesion. Generally, most grading systems categorize tumors into three or four grades based on cellular differentiation [53], whereby high-grade cancers are more poorly differentiated and clinically aggressive than low-grade cancers. Histopathologic grading is as crucial as anatomic staging to predict patient prognosis and guide treatments [53]. Thus, biopsy or excision of suspicious lesions is important for cancer diagnosis and classification of tumor cellular architecture. However, the subjective histopathological diagnosis is invasive and often leads to misdiagnosis [3].

An essential potential application of FTIR spectroscopy is its use in accurate cancer classification, staging and grading. *Lima* et al. [16] have achieved up to 100% of sensitivity and specificity that is necessary for real-world ovarian cancer diagnosis using ATR-FTIR spectroscopy combined with successive projection algorithm, variable selection methods or genetic algorithm with linear discriminant analysis (GA-LDA). The study demonstrated the accurate diagnosis for different ovarian cancer stages and histological type as well as differentiation based on age using plasma and serum specimens, justifying that the technique is particularly useful for biomarker discovery and a potential population-based screening tool for ovarian cancer [16]. On the other hand, Baker et al. [20] investigated FTIR-based histopathology for prostate cancer diagnosis. FTIR microspectroscopy coupled with principal component—discriminant function analysis (PC-DFA) was used to analyze formalin-fixed archival prostate cancer tissues. The authors examined the spectral signatures that identify subtypes of prostate cancer and correlate to the observer dependent criterion of Gleason grading as well as the observer independent TNM staging system. The Gleason grade is a qualitative evaluation of the loss of normal glandular prostate tissue architecture by observing a stained prostate tissue section [54,55], with higher scores showing greater loss of the normal glandular morphology. The study revealed the ability of the technique to discriminate biochemical changes of the disease and that the spectral signatures could differentiate confined prostate cancer from the invasive ones, demonstrating sensitivity and specificity as high as 83.6% and 86.0% respectively for the method to distinguish prostate cancer tissues based on the Gleason criterion [20].

While the current gold standard for most cancer diagnosis remains as the invasive histopathological diagnosis, this subjective and time-consuming method warrants novel diagnostic techniques with improved accuracy in classification, staging and grading for cancer management. Due to the unique anatomical spread patterns of cancer, each cancer type may require distinct classification system. FTIR spectroscopy may represent a better technique as it could provide specific spectral signatures for each unique disease, including each different stage and grade of each cancer type. Moreover, many investigations on the application of FTIR spectroscopy have demonstrated its strong potential in this important clinical need, which may substantially enhance cancer management and improve patient care.

## 5. Automated Cancer Diagnosis

In this era of technological advancement, automation has arisen in many different fields, including the diagnosis of cancer. Following the reduced cost of electronic components, computers with enhanced processing capabilities and memory capacity are built, leading to the rise of computer aided/assisted diagnosis (CAD) which combines algorithms or methods from pattern recognition and digital image processing [56]. As histopathology study is considered as the current gold standard in cancer diagnosis, computers have been applied to interpret histopathology images to aid pathologists during the diagnosis process. Subsequently, this allows diagnosis procedures to become reliable, reproducible and less subjected to observer variations. Nevertheless, tissue biopsy samples of patients obtained during surgical procedure have to be clinically processed through fixation, dehydration, clearing, infiltration, embedding, sectioning and staining before acquiring histopathology images using hardware devices, such as the microscope, camera or slide scanners [57].

Importantly, the application of automated FTIR spectroscopy in clinical diagnostic settings has been investigated to improve accuracy and reproducibility of cancer diagnosis, while omitting the need for complex and time-consuming clinical processing of tissue biopsy samples. Automated marker-free histopathological annotation of lung tumor classes and subtypes of adenocarcinoma without further treatment of the tissue samples have been conducted by Großerueschkamp et al. [21] using FTIR imaging and a novel trained random forest (RF) classifier. Clinical histopathology of fresh frozen lung tissue samples were evaluated and meta data of patients, such as smoking status and pre-treatment, were considered in the analysis for the challenging subtyping of the highly inherent histologically heterogeneous lung tumor [58,59]. The results showed greater reproducibility as well as high accuracy of 97% for the annotation of lung tumor classes and 95% for the identification of prognostic adenocarcinoma subtypes, respectively. Importantly, the automated FTIR technique reduced intra- and inter-operator variability through its objectivity, reproducibility and improved accuracy over current methodologies for lung tumor diagnosis.

Many of the current automated image analysis systems aim to detect certain objects of interest, such as glands, lymphocytes, mitosis and nuclei, among the various elements found in a histopathology image. However, challenges including out-of-focus objects, missing or broken boundaries of objects, variations in shape and size of objects, differences of intensity levels within objects, similarity between objects of interest and other artefacts as well as overlapping structures, may hinder this task [60]. Additionally, variations in image acquisition conditions and tissue preparation process commonly cause differences in the acquired images. Compared with hematoxylin and eosin (H&E) stained histological images, index color image acquired from automated FTIR imaging reflects the morphology much more precisely [21]. Furthermore, high reproducibility is achieved as errors from manual handling were excluded by automation of sample preparation and spectrum processing. This permits the standardization of sample preparation as well as spectral measurement and analysis which will be necessary for the construction of FTIR spectral databases with highly specific spectroscopic markers for the various stages and grades of different cancer types in order to apply the technique in clinical settings [61,62]. Hence, FTIR spectral histopathology would be crucial for the acceleration of point-of-care decisions and improvement of therapy decisions in personalized medicine. With the spectral database, the approach could provide the advantage of being able to screen multiple types of cancer as a stand-alone tool in contrast with majority of existing laboratory tests that focus on the detection of a single cancer type [63]. Additionally, in the attempt to progress FTIR spectroscopy from “bench top to bedside”, novel powerful algorithms for automatic data analysis of large data sets have been developed to enable easy and objective data interpretation by non-spectroscopists.

## 6. Cancer Surgical Management

Chemotherapy, radiotherapy and surgery are the most common types of cancer treatments nowadays, with surgery being the basis for solid tumor treatment. To ensure complete removal of tumor during surgery, visible tumor is resected with a fringe of normal tissue. Adequacy of resection margins in surgery is of utmost importance to prevent under- or over-treatment as the range of surgical removal is directly correlated to long-term survival and post-operative recovery of patients. Overly small resection range may cause tumor recurrence after surgery and affect the long-term survival, while a beyond range of resection could result in longer duration of postoperative recovery or even surgical complications [24]. However, this is mainly based on the interpretation of imaging investigations, preoperative planning of the resection extent and clinical judgment of the surgeon [64]. Resected tumor specimens are examined by surgical pathologist to determine whether the tumor is entirely excised. The examination is performed intra-operatively for consultation and post-operatively for routine pathological assessment. Nevertheless, intraoperative microscopic evaluation is performed on frozen tissue sections, whereby only few margins nearest to the tumor can be examined due to the limited time available [64]. Furthermore, inherent artefacts such as thawing of sections and ice crystals formation may affect the microscopic appearance of the tissue specimens and cause difficulties in interpretation.

Depciuch et al. demonstrated both Raman spectroscopy and IR spectroscopy could detect changes of various biomolecules in breast cancer tissues in comparison with the normal samples [65]. Further, FTIR spectrometry offers rapid and objective diagnosis of tumors to assist surgical decision making. Yao et al. [24] have evaluated the surgical resection margin during surgery of colorectal cancer using ATR-FTIR spectroscopy combined with optical fiber. Colorectal tumor as well as mucosa 1, 2 and 5 cm from the tumor were examined with the technique. Intriguingly, the spectra of colorectal tumor and mucosa 1 cm away from it were different than those of 2 and 5 cm from the tumor, signifying a promising intraoperative and rapid diagnostic method to judge the safety of surgical resection margin for colorectal cancer. In future, investigations with the integration of flexible optic fiber to FTIR spectroscopy would allow the in vivo real time pre- or intra-operative diagnosis of cancer that can guide surgeons to avoid unnecessary dissection and minimize surgical trauma [66,67]. Furthermore, early detection of colorectal cancer relapse and assessment of colon tissue abnormality were reported by Salman et al. [23] using FTIR spectroscopy as well as principal component analysis (PCA) and linear discriminant analysis (LDA) to accurately determine margins of tumor and reduce recurrences. Colorectal tissues from control, local and distant recurrence crypts were evaluated and the spectra measured resulted with high success rate of more than 92% for differentiation between the specimens. This study signifies the ability of FTIR spectroscopy in aiding surgeons to determine the presence of metastatic and recurrence potential in resection margins which would lead to better prognosis for patients.

Approximately 40 min are needed for intraoperative pathological examination of frozen section, which is the most common method used to assess the cutting edge [24]. Multiple such examinations may also be required during the operation when interpretation for the diagnosis is difficult. Therefore, this method is subjective to the surgical pathologist’s judgement and would prolong the operation time that could cause adverse effects on the postoperative recovery of patients. Meanwhile, much lesser time is required for each FTIR measurement, which takes only around one to three min [24]. Moreover, FTIR spectroscopy permits the detection of early stage abnormality at the molecular level of resection margins when the morphology is still normal, which could not be achieved in standard pathology tests and subsequently led to high local recurrence rate [23]. Thus, the novel technique provides a rapid and objective method to detect cancer for personalized intra- and post-operative management whilst reducing resource expenditure and surgery-related risks to patients. Additionally, coupling FTIR spectroscopy with technologies such as optical fiber would enable in vivo real time, non-invasive cancer diagnosis which could determine safety of resection margins and prevent unnecessary dissection as well as reduce surgical trauma [66,67].

## 7. Monitoring of Cancer Treatment Response and Follow-Up

Besides surgery, chemotherapy and radiotherapy which have been the most widely used treatments, immunotherapy has become an essential therapeutic alternative in recent years and is now the treatment of choice in many cases. On the other hand, nanotechnology has lately offered nanostructures as novel therapeutic options with functions to enhance treatment outcome, such as controlled drug delivery, directed target therapy and treatment combination [68]. Subsequently, monitoring of treatment response is essential for cancer management and treatment planning in personalized medicine to increase survival chances. While the improved therapeutic options have resulted in better survival rates for cancer patients, recurrences still occur and cancer survivors are often affected by symptoms, side effects and psychological concerns due to the treatments [69,70]. Survival statistics are the most common measures used to predict the prognosis of cancer patients and their likely course of disease. Consequently, follow-up after cancer treatments are important for early detection of recurrences and secondary tumors, treatment of side effects from therapy as well as support for mental or psychosocial stress in patients [71]. General assessments used in treatment monitoring and follow-up include routine blood and/or laboratory tests, imaging procedures, sonographic examinations and determination of tumor markers, such as the prostate-specific antigen (PSA) [72], which are also the currently used cancer diagnostic methods that can be time consuming and lack the high sensitivity and/or specificity as mentioned in previous section (Section “Sensitivity, specificity and accuracy in cancer detection”).

An important feature of FTIR spectroscopy is its ability to detect the presence of relapse as well as to monitor therapeutic efficacy in patients. Kaznowska et al. [22] investigated the application of FTIR spectroscopy and principal component analysis-linear discriminant analysis (PCA-LDA) to detect spectral differences between colon tissues from healthy colon, surgical margin of colorectal tumor as well as cancerous pre- and post-chemotherapy colon, which could facilitate pathophysiological interpretation of various conditions and monitoring of chemotherapy efficacy. Interestingly, spectral analysis revealed differences between each type of tissue specimen and comparison between healthy and post-chemotherapy colon tissues suggests the potential for assessing treatment efficacy whereby higher degree of similarity between the two types of tissues indicates greater effectiveness. Additionally, the study also demonstrated FTIR spectroscopy as a prospective tool to define margin of the tumor, inclusive of even single cancer cells, before resection procedures which can increase survival chances of patients [22].

Nevertheless, given that blood samples can be obtained less invasively, the use of these specimens may be more favorable compared to tissue samples for the monitoring of therapeutic response and disease progression. Zelig et al. [19] have utilized identified diagnostic markers from peripheral blood mononuclear cells of childhood acute leukemia patients using FTIR microspectroscopy to monitor the disease during chemotherapy treatment. Pre-screening and long term follow-up of the disease were conducted using blood samples from leukemia patients before and during the treatment, while blood samples from healthy subjects and patients with infection who exhibited “flu-like” clinical symptoms similar with leukemia acted as control groups. This clinical study demonstrated the application of FTIR microspectroscopy with cluster analysis (CA) for pre-screening independently of symptoms common with leukemia. Thus, the study proved the significant potential of FTIR microspectroscopy as a complementary tool for rapid leukemia pre-screening and follow-up to provide precursor indication of patient response to chemotherapy compared with conventional methods for sooner response to critical complications and improve treatment management [19].

From the perspectives of both cancer patients and physicians, the main purpose in follow-up is the early detection of recurrences [73]. However, existing methods for follow-up lack the sensitivity and specificity for effective early detection of recurrences. It was revealed that only 40% of isolated locoregional recurrences were detected in asymptomatic patients during routine examinations in a meta-analysis of more than 5000 patients [74]. Therefore, the application of the highly sensitive and specific FTIR spectroscopy could improve the follow-up of cancer patients, especially in the early detection of recurrences. In addition, the approach could enable rapid analysis of patients’ samples for sooner response to critical conditions during treatment when compared to the time-consuming tests currently used in cancer management.

## 8. Fourier Transform Infrared Spectroscopic Analysis of Cancer-Derived Extracellular Vesicles

### 8.1. Diagnostic Value of Extracellular Vesicles

Extracellular vesicles (EVs) are cell-derived membrane nanovesicles that are released to the extracellular space and circulation. They contribute to intercellular communication and reflect the physiological as well as pathological conditions in the body [34]. EVs have recently gained the attention of researchers in the field of clinical research. These vesicles are a heterogenous population of particles varying between 30–1000 nm in diameter that are classified into a few subsets based on their size, density, morphology, biogenesis, origin, lipid composition, sedimentation characteristics and biochemical markers specifically present on their surface [75,76]. These subsets include exosomes (30–100 nm), microvesicles and late endosomes (50–1000 nm), ectosomes (100–350 nm) as well as microparticles (100–1000 nm). Additionally, several apoptotic bodies (0.5–5 μm) and “small-size microparticles” (<50 nm) are also grouped as EVs.

Recent studies suggest that EVs are the transport form for various molecules such as mRNAs, miRNAs, cytokines, hormones, autoantigens, surface receptors and tissue coagulation factors, which might be paracrine regulators of target cell function and metabolism from their parental cells [77,78,79]. Furthermore, the biological molecules encompassed in EVs are involved in diverse processes including proliferation, malignancy, vasculogenesis, inflammation, infections, tissue repair as well as growth and differentiation of tissues [80,81]. EVs contribute to disease progression by modulating local and systemic effects in the body. In fact, EVs have been identified in many biological tissues and fluids, including blood and saliva, as targets of treatments and biomarkers of diseases [82,83]. Studies have found that the characteristic morphological and molecular features of salivary exosomes of oral cancer patients were different from that of healthy individuals [84,85]. To date, numerous evidences are present suggesting the use of EVs in diagnosis with promising predictive value in various diseases including cancer [86,87], diabetes [88], cardiovascular [89,90] and autoimmune [91] diseases as well as central nervous system disorders [92]. Being released into bodily fluids, EVs could serve in nanomedicine as an invaluable source of non-invasive diagnostic specimen that is simpler and yet representative of the pathophysiological conditions in the body which overcome the limitations of the commonly used blood and tissue samples [93]. Moreover, EVs can provide diagnostic information at successive time points for early detection and monitoring of both local and systemic diseases.

### 8.2. Analysis of Extracellular Vesicles Using Fourier Transform Infrared Spectroscopy

Despite numerous studies demonstrating the potential use of EVs in disease diagnosis, FTIR spectroscopic analysis of EVs for cancer diagnosis has not been widely investigated. One of such investigation is from Krafft et al. [25] who have examined the diagnostic value of EVs in the screening of prostate cancer by using FTIR and Raman spectroscopy to perform a comprehensive comparative analysis between cancer and non-cancer EVs. Differential centrifugation of plasma and serum from patients with prostate cancer or benign prostatic hyperplasia as well as a healthy donor isolated two distinct EV fractions enriched with microvesicles and exosomes, which are the most abundant and well-investigated EVs. Cancer spectral signature of the blood EVs was utilized in a pilot study to detect prostate cancer from a test cohort of patients, including four with benign prostatic hyperplasia and another four with high-grade prostate carcinoma. The study concluded that the identified EV signature is a useful screening tool in cancer detection. Notably, the authors demonstrated that the isolation of EV fraction as an analyte is necessary because both the IR and Raman data resulted from direct analysis of unprocessed serum and plasma from cancer and healthy donors were almost identical and are insufficient to detect changes for cancer screening. Samples are usually dried before spectroscopic analysis to avoid the strong spectral contributions from water in the specimen from masking the spectrum differences. Hence, broad variations in the study observed between 1200 and 1400 cm^−1^ from liquid but not in dry phases of the serum and plasma samples were considered irrelevant [25].

**Figure 1:**
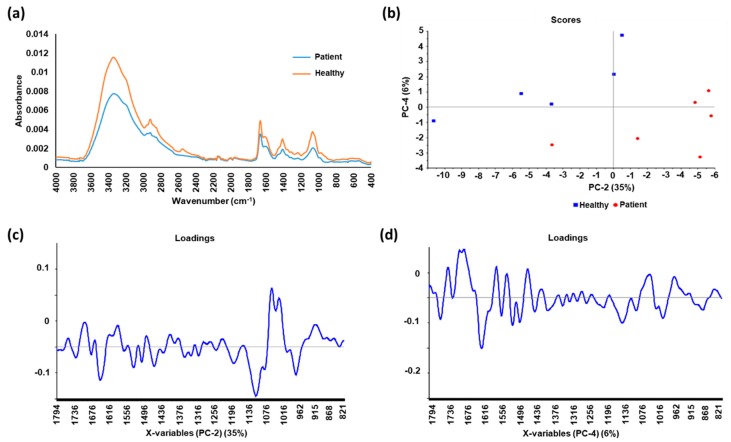
The average spectra and principal component analysis (PCA) of urinary extracellular vesicle (EV) samples from prostate cancer patients and healthy individuals (**a**) The average spectra of the EV samples from patients has a lower absorbance compared to that from healthy individuals; (**b**) Score plot of second and fourth PCs, with corresponding percentage of explained variance in parentheses; (**c**) Loadings of the second PC with 35% of explained variance; (**d**) Loadings of the fourth PC with 6% of explained variance. Spectral peak differences between the two groups were revealed after analyzing the spectra with PCA, suggesting possible biomarkers for prostate cancer. Linear discriminant analysis (LDA) was used to derive a diagnostic classifier for prostate cancer from the spectra, which achieved sensitivity of 83.33% and specificity of 60%.

In another study, Zlotogorski-Hurvitz et al. [27] assessed the diagnostic potential of salivary exosomes for early detection of oral cancer using ATR-FTIR spectroscopy and machine learning techniques, including principal component analysis-linear discriminant analysis (PCA-LDA) and support vector machine (SVM) classification. Exosomes were isolated from whole saliva samples of oral cancer patients and healthy individuals using differential centrifugation. The findings showed that the IR spectra were consistently different between the two groups and that specific spectral signature for the cancer salivary exosomes was accurately distinguished from exosomes of healthy individuals. Furthermore, classification of samples resulted with high sensitivity, specificity and accuracy of 100%, 89% and 95%, respectively. Recently, we have coupled ATR-FTIR analysis of urinary EVs with PCA-LDA statistic model (Figure 1) in our laboratory as a novel strategy for non-invasive early detection of prostate cancer [26]. The spectral differences between the EVs from prostate cancer patients and healthy individuals as well as the analysis using linear discriminant analysis (LDA)-derived classifier, which achieved sensitivity of 83.33% and specificity of 60%, signifies the potential of ATR-FTIR technique as a point-of-care test for prostate cancer in urine.

The ability of FTIR spectroscopy to provide accurate signature of biomolecular content of the analyte with small-scale preparation and high-speed detection, allows the development of a label-free disease-specific EV biomarker which is both time and cost-effective. However, the current most commonly used method in EV studies, differential centrifugation, only allows isolation of enriched fraction of the EV subtypes. Therefore, development of novel methodology for complete purification of each EV subtype would be required to aid in cancer biomarker identification as well as for the study of EVs and their role in cancer, which are still poorly understood. Furthermore, it is important to note that the promising findings of FTIR spectroscopy warrants further research for validation in larger patient cohorts with varying stages and different grades of cancer for clinical translation of the methodology.

## 9. Challenges and Future Directions of Fourier Transform Infrared Spectroscopy in Clinical Use

As more studies show promising results of FTIR spectroscopic analysis using biological specimens in cancer screening, diagnosis, management and monitoring, the challenge now is to translate these methods to routine clinical practice. Nonetheless, most of these studies have been performed on rather small sample size. Hence, large scale clinical trials are required to prove its utility in actual clinical environment and illuminate barriers in implementation that need to be overcome. Large patient cohorts with varying stages and different grades of cancer will be useful in validating the technique. Appropriate selection of patients and control subjects is of utmost importance to reduce the risk of false positives as unmatched comparison groups in sex, age, and physical conditions including hormonal status or pathologies besides the disease of interest, may lead to biased results and differences between the groups could be caused by these confounding factors instead of the disease of interest [94].

Furthermore, standardization of sample collection and storage is vital to not only achieve experimental reproducibility in an individual laboratory but among different laboratories as well [95]. To date, research in the application of FTIR spectroscopy in the study of cancer involves the analysis of many different types of biological materials, including the most commonly analyzed specimens, human tissues and bodily fluids. However, the spectroscopic study of each of these biological materials has its own challenges. For instance, scattering artefacts may occur when tissue samples are analyzed using transmission-mode FTIR spectroscopy. Meanwhile, drying of drop deposits commonly performed to measure biofluids using the ATR mode gives rise to heterogeneous drop deposition, which is characterized by the well-known coffee-ring effect. The effect is resulted from migration of macromolecules towards periphery of the drop [96,97,98]. Thus, strict control of experimental parameters for drop deposition is necessary to attain reproducible results [99]. This could be achieved by automated sampling approach as depicted by Ollesch et al. [61], who have reported higher reproducibility in spectral data compared to non-automated sampling. Hence, the necessity of automated instruments highlights the need for a close collaboration among research scientists, industrial partners and clinical practitioners to optimize existing products based on a specific biomedical purpose [100].

Although FTIR spectroscopic analyses of many types of biological materials have been shown to be possible methods for clinical applications in cancer, there are limitations of using these complex biological specimens. Firstly, the use of the current commonly analyzed blood and tissue samples are collected invasively from patients and requires the need for trained medical personnel which would greatly increase the cost and time required for diagnosis. Additionally, contamination during sample preparation such as paraffin in tissue specimens may produce background and interfere with the spectral information [101]. Although the use of paraffin in tissue sample preparation has been shown to have no effect on FTIR spectroscopic results [101,102], the immense biological variability within these complex specimens causes large background absorbance that may mask the small specific spectral changes and hinder the identification of biomarkers. Likewise, the strong interfering spectroscopic signal from the erythrocyte component in blood samples may potentially mask the underlying changes of clinically relevant biomolecules observed in cancer versus non-cancer patients [103]. Hence, examining extracellular vesicles (EVs) allows rapid, inexpensive and non-invasive diagnosis with high sensitivity, specificity and accuracy, which eliminates the abundant uninformative data for more effective biomarker identification. In our laboratory, a methodology to study prostate cancer-derived EVs in urine using ATR-FTIR spectroscopy has been established [26]. Nonetheless, development of novel standardized methodology to isolate each EV subtype may be useful to aid cancer biomarker identification and improve the diagnostic method.

Another factor to be considered for the implementation of FTIR spectroscopy for clinical use is that instruments from different manufacturers may have distinct responses and spectral distortions. Moreover, backgrounds have to be addressed using pre-processing algorithms in order to compare results between different studies. Besides that, considerations on the method of sample preparation as well as the optical substrate and acquisition mode used should be taken into account in pre- and post-processing procedures [95]. Additionally, technical standardization of spectral acquisition may allow reproducible results to be obtained in different laboratories and the external validation will be important for clinical validity [95]. Several multivariate approaches have been utilized to build classification models, yet there is no consensus on the best method to date. Notably, the classifier outcome can be impacted by small dataset that does not correctly define the patient population and result in under- or over-fitting [95]. Therefore, it is essential to have a large amount of class-representative patient samples for a classifier to be robust. In contrast to classical statistics, no simple method is available to calculate sample size for biospectroscopic studies. Nevertheless, Beleites et al. [104] have proposed the use of learning curves to determine suitable sample size required to build good classifiers with specified performances. Finally, the advantages of using the spectroscopic biomarker in clinical decision-making setting and its favorable medico-economic profile should be clearly demonstrated to accomplish routine clinical use [105].

## 10. Conclusions

Taken together, FTIR spectroscopy holds promise for use as a novel clinical tool for cancer. Importantly, this is evident through the numerous investigations using the technique on multiple cancer types (Table 2). The technique offers high sensitivity, specificity and accuracy in cancer detection when compared to currently used diagnostic methods. Furthermore, FTIR spectroscopy enables accurate and objective classification, staging and grading for cancer management as opposed to the present gold standard, histopathological diagnosis, to guide treatments and predict patient prognosis. Notably, automated marker-free FTIR spectroscopy allows higher accuracy and reproducibility in cancer diagnosis, while eliminating the need of complex and time-consuming clinical processing of tissue samples required in existing computer-aided histopathological diagnosis. In addition, it has been demonstrated that FTIR spectroscopy could be used to evaluate surgical resection margins rapidly and objectively to assist surgical decision making which will improve long-term survival and postoperative recovery of patients when compared to the common intraoperative pathological examination. The technique has also been utilized to monitor cancer treatment response and follow-up of patients for treatment planning, early detection of recurrences and support for mental or psychosocial stress with more rapid, sensitive and specific results as compared to current methods. Hence, FTIR spectroscopy would be crucial to accelerate point-of-care decisions and potentially revolutionize cancer diagnostics in personalized medicine.

Even though FTIR spectroscopy may not be able to identify specific molecules when compared to molecular tests, it allows the qualitative and quantitative analyses of different classes of molecules simultaneously such as nucleic acids, proteins and lipids. Therefore, the technique provides the overall status of the analyzed specimen and is ideal for complicated diseases like cancer, which are multifactorial and examining isolated molecules alone may not give complete information [107]. Hence, this feature provides the advantage of being able to reveal the whole “omics” of the examined specimen and identify more than one cancer biomarker. Additionally, the comprehensive information of patients provided by FTIR spectroscopy also reveals new insights in cancer research by allowing better understanding of mechanisms underlying carcinogenesis, such as the significant quantitative changes in spectral regions of nucleic acids and proteins indicate metabolic dysfunction in cancer cells and can be considered as biomarkers [22]. Interestingly, Kyriakidou et al. [106] have reported spectroscopic data showing melanoma alters the permeability of cell membrane and changes the native B-DNA form to Z-DNA form, suggesting possible biomarker for skin cancer while further understanding the biochemical changes in the disease. The promising findings of FTIR spectroscopy in cancer screening, diagnosis, management and monitoring demonstrates its value for further research and development. Besides validating the technique in larger patient cohorts, challenges in technological development, standardization of sample collection, storage and preparation methods, data acquisition procedures, pre- and post-processing of spectral data as well as classification models will have to be overcome for successful clinical translation.

## Figures and Tables

**Table 1:** Assignment of typical absorption bands observed in biological IR spectra.

Wavenumber (cm^−1^)	Assignment
3080–2800	Anti-symmetric and symmetric C–H stretches from proteins and lipids
1745–1725	Ester carbonyl of lipids
1700–1500	Amide I and II groups in peptide linkages of proteins
1270–1080	Anti-symmetric and symmetric C−O and P−O areas in DNA, RNA and phospholipids
1200–900	Carbohydrate vibrations of glucose, fructose and glycogen

**Table 2:** Studies of various cancer types using FTIR spectroscopy discussed in this review article.

Cancer Type	Title of Study	References
**Colorectal Cancer**	Application of linear discriminant analysis and attenuated total reflectance Fourier transform infrared microspectroscopy for diagnosis of colon cancer	[4]
	The use of FTIR-ATR spectrometry for evaluation of surgical resection margin in colorectal cancer: a pilot study of 56 samples	[24]
	Early detection of colorectal cancer relapse by infrared spectroscopy in “normal” anastomosis tissue	[23]
	Use of FTIR spectroscopy and PCA-LDC analysis to identify cancerous lesions within the human colon	[22]
**Prostate Cancer**	Study of prostate cancer-derived extracellular vesicles in urine using IR spectroscopy	[26]
	Investigating FTIR based histopathology for the diagnosis of prostate cancer	[20]
	A specific spectral signature of serum and plasma-derived extracellular vesicles for cancer screening	[25]
**Leukemia**	Distinction of leukemia patients’ and healthy persons’ serum using FTIR spectroscopy	[17]
	Pre-screening and follow-up of childhood acute leukemia using biochemical infrared analysis of peripheral blood mononuclear cells	[19]
**Ovarian and/or Endometrial Cancers**	Potential of mid-infrared spectroscopy as a non-invasive diagnostic test in urine for endometrial or ovarian cancer	[28]
	Segregation of ovarian cancer stage exploiting spectral biomarkers derived from blood plasma or serum analysis: ATR-FTIR spectroscopy coupled with variable selection methods	[16]
**Lung Cancer**	Evaluation of FTIR spectroscopy as a diagnostic tool for lung cancer using sputum	[31]
	Marker-free automated histopathological annotation of lung tumor subtypes by FTIR imaging	[21]
**Oral, Oropharyngeal, and/or Laryngeal Cancer**	Fourier transform infrared for noninvasive optical diagnosis of oral, oropharyngeal, and laryngeal cancer	[32]
	FTIR-based spectrum of salivary exosomes coupled with computational-aided discriminating analysis in the diagnosis of oral cancer	[27]
**Gastric Cancer**	Comparison of serum from gastric cancer patients and from healthy persons using FTIR spectroscopy	[18]
**Breast Cancer**	Diagnosis of breast cancer with infrared spectroscopy from serum samples	[15]
**Bladder Cancer**	Bladder cancer diagnosis from bladder wash by Fourier transform infrared spectroscopy as a novel test for tumor recurrence	[29]
**Malignant** **Biliary Strictures**	Bile analysis using high-throughput FTIR spectroscopy for the diagnosis of malignant biliary strictures: a pilot study in 57 patients	[30]
**Skin Cancer**	FT-IR spectroscopy study in early diagnosis of skin cancer	[106]
